# Bis(benzyl­triethyl­ammonium) hexa­chloridostannate(IV)

**DOI:** 10.1107/S1600536810038122

**Published:** 2010-09-30

**Authors:** Ezzatollah Najafi, Mostafa M. Amini, Seik Weng Ng

**Affiliations:** a Department of Chemistry, General Campus, Shahid Beheshti University, Tehran 1983963113, Iran; b Department of Chemistry, University of Malaya, 50603 Kuala Lumpur, Malaysia

## Abstract

The reaction between benzyl­triethyl­ammonium chloride and dimethyl­tin dichloride yields the title salt, [(C_6_H_5_CH_2_)(C_2_H_5_)_3_N]_2_[SnCl_6_]. The Sn^IV^ atom, located on a center of inversion, exists in an octa­hedral coordination environment. The cation links with the anion *via* weak C—H⋯Cl hydrogen bonding.

## Related literature

For bis­(tetra­methyl­ammonium) hexa­chloridostannate(IV), see: Furukawa *et al.* (1982 ▶). For bis­(tetra-*n*-propyl­ammonium) hexa­chloridostannate(IV), see: James *et al.* (1992 ▶). For bis­(tetraethyl­ammonium) hexa­chloridostannate(IV), see: Sowa *et al.* (1981 ▶).
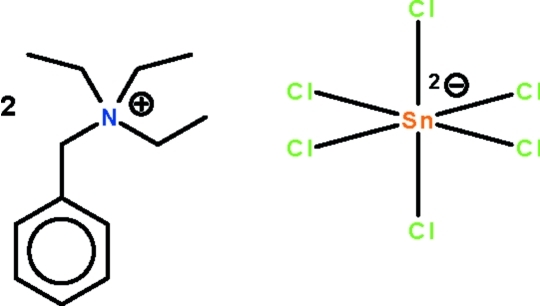


## Experimental

### 

#### Crystal data

(C_13_H_22_N)_2_[SnCl_6_]
*M* *_r_* = 716.02
Monoclinic, 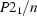

*a* = 11.2096 (6) Å
*b* = 11.2306 (6) Å
*c* = 12.9796 (7) Å
β = 90.872 (1)°
*V* = 1633.82 (15) Å^3^
*Z* = 2
Mo *K*α radiation
μ = 1.29 mm^−1^
*T* = 295 K
0.30 × 0.20 × 0.10 mm

#### Data collection

Bruker SMART APEX diffractometer
Absorption correction: multi-scan (*SADABS*; Sheldrick, 1996 ▶) *T*_min_ = 0.698, *T*_max_ = 0.882
15028 measured reflections
3756 independent reflections
3276 reflections with *I* > 2σ(*I*)
*R*_int_ = 0.022

#### Refinement

*R*[*F* ^2^ > 2σ(*F* ^2^)] = 0.021
*wR*(*F* ^2^) = 0.059
*S* = 1.01
3756 reflections
160 parameters
H-atom parameters constrained
Δρ_max_ = 0.41 e Å^−3^
Δρ_min_ = −0.39 e Å^−3^

### 

Data collection: *APEX2* (Bruker, 2009 ▶); cell refinement: *SAINT* (Bruker, 2009 ▶); data reduction: *SAINT*; program(s) used to solve structure: *SHELXS97* (Sheldrick, 2008 ▶); program(s) used to refine structure: *SHELXL97* (Sheldrick, 2008 ▶); molecular graphics: *X-SEED* (Barbour, 2001 ▶); software used to prepare material for publication: *publCIF* (Westrip, 2010 ▶).

## Supplementary Material

Crystal structure: contains datablocks global, I. DOI: 10.1107/S1600536810038122/xu5035sup1.cif

Structure factors: contains datablocks I. DOI: 10.1107/S1600536810038122/xu5035Isup2.hkl

Additional supplementary materials:  crystallographic information; 3D view; checkCIF report

## Figures and Tables

**Table 1:** Hydrogen-bond geometry (Å, °)

*D*—H⋯*A*	*D*—H	H⋯*A*	*D*⋯*A*	*D*—H⋯*A*
C2—H2*B*⋯Cl1^i^	0.96	2.74	3.685 (3)	169

Symmetry code: (i) 
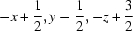
.

**Fig. 1.**
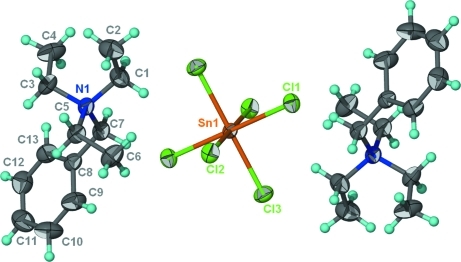
Thermal ellipsoid plot (Barbour, 2001) of the 2(C6H5CH2)(C2H5)3N+ SnCl62- salt at the 50% probability level. Hydrogen atoms are drawn as spheres of arbitrary radius.

## supplementary crystallographic information

### Comment

The reaction of dimethyltin dichloride with ammonium halides sometimes leads to tin-carbon cleave to result in the formation of a hexahalogenostannate. Tin-methyl cleavage was noted in the reaction of dimethyltin dichloride with and benzyltriethylammonium chloride; the resulting the title salt (Scheme I, Fig. 1) consists of ammonium cations and hexachloridostannate anions. The reported ammonium hexachloridostannates all have symmetrically substituted ammonium cations.

### Experimental

Dimethyltin(IV) dichloride (0.219 g, 1 mmol) and benzyltriethylammonium chloride (0.455 g, 2 mmol) were dissolved in methanol and the solution kept at 333 K. Crystals were isolated after several days; m.p. 452–454 K.

### Refinement

Hydrogen atoms were placed in calculated positions (C–H 0.93–0.97 Å) and were included in the refinement in the riding model approximation, with *U*(H) set to 1.2–1.5*U*_eq_(C).

### Crystal data

**Table:**

(C_13_H_22_N)_2_[SnCl_6_]	*F*(000) = 732
*M**_r_* = 716.02	*D*_x_ = 1.455 Mg m^−^^3^
Monoclinic, *P*2_1_/*n*	Mo *K*α radiation, λ = 0.71073 Å
Hall symbol: -P 2yn	Cell parameters from 8245 reflections
*a* = 11.2096 (6) Å	θ = 2.4–28.2°
*b* = 11.2306 (6) Å	µ = 1.29 mm^−^^1^
*c* = 12.9796 (7) Å	*T* = 295 K
β = 90.872 (1)°	Prism, colorless
*V* = 1633.82 (15) Å^3^	0.30 × 0.20 × 0.10 mm
*Z* = 2	

### Data collection

**Table:**

Bruker SMART APEX diffractometer	3756 independent reflections
Radiation source: fine-focus sealed tube	3276 reflections with *I* > 2σ(*I*)
graphite	*R*_int_ = 0.022
ω scans	θ_max_ = 27.5°, θ_min_ = 2.4°
Absorption correction: multi-scan (*SADABS*; Sheldrick, 1996)	*h* = −14→13
*T*_min_ = 0.698, *T*_max_ = 0.882	*k* = −14→14
15028 measured reflections	*l* = −16→16

### Refinement

**Table:**

Refinement on *F*^2^	Primary atom site location: structure-invariant direct methods
Least-squares matrix: full	Secondary atom site location: difference Fourier map
*R*[*F*^2^ > 2σ(*F*^2^)] = 0.021	Hydrogen site location: inferred from neighbouring sites
*wR*(*F*^2^) = 0.059	H-atom parameters constrained
*S* = 1.01	*w* = 1/[σ^2^(*F*_o_^2^) + (0.032*P*)^2^ + 0.4848*P*] where *P* = (*F*_o_^2^ + 2*F*_c_^2^)/3
3756 reflections	(Δ/σ)_max_ = 0.001
160 parameters	Δρ_max_ = 0.41 e Å^−^^3^
0 restraints	Δρ_min_ = −0.39 e Å^−^^3^

### Fractional atomic coordinates and isotropic or equivalent isotropic displacement parameters (Å^2^)

**Table:**

	*x*	*y*	*z*	*U*_iso_*/*U*_eq_	
Sn1	0.5000	0.5000	0.5000	0.03041 (6)	
Cl1	0.53546 (4)	0.67877 (4)	0.60050 (3)	0.04330 (11)	
Cl2	0.45054 (4)	0.39111 (4)	0.65796 (3)	0.04483 (12)	
Cl3	0.70731 (4)	0.44472 (5)	0.52383 (4)	0.04963 (13)	
N1	0.10908 (13)	0.22025 (14)	0.56788 (12)	0.0390 (3)	
C1	0.1030 (2)	0.3475 (2)	0.60638 (18)	0.0534 (5)	
H1A	0.1762	0.3649	0.6443	0.064*	
H1B	0.0997	0.4002	0.5472	0.064*	
C2	−0.0015 (2)	0.3758 (2)	0.6748 (2)	0.0659 (7)	
H2A	0.0027	0.4576	0.6958	0.099*	
H2B	0.0013	0.3254	0.7345	0.099*	
H2C	−0.0748	0.3622	0.6373	0.099*	
C3	−0.00149 (17)	0.1864 (2)	0.50650 (16)	0.0519 (5)	
H3A	−0.0680	0.1816	0.5533	0.062*	
H3B	0.0103	0.1075	0.4779	0.062*	
C4	−0.0354 (2)	0.2704 (3)	0.41919 (19)	0.0744 (8)	
H4A	−0.1066	0.2421	0.3853	0.112*	
H4B	0.0283	0.2736	0.3707	0.112*	
H4C	−0.0492	0.3486	0.4464	0.112*	
C5	0.11816 (18)	0.13332 (19)	0.65726 (15)	0.0460 (5)	
H5A	0.1295	0.0539	0.6296	0.055*	
H5B	0.0429	0.1336	0.6932	0.055*	
C6	0.2178 (2)	0.1584 (3)	0.73500 (17)	0.0627 (6)	
H6A	0.2181	0.0979	0.7873	0.094*	
H6B	0.2051	0.2348	0.7662	0.094*	
H6C	0.2930	0.1583	0.7007	0.094*	
C7	0.22048 (17)	0.21310 (18)	0.50080 (15)	0.0436 (4)	
H7A	0.2122	0.2710	0.4457	0.052*	
H7B	0.2891	0.2363	0.5426	0.052*	
C8	0.24596 (17)	0.09404 (19)	0.45360 (15)	0.0429 (4)	
C9	0.3185 (3)	0.0112 (2)	0.5036 (2)	0.0660 (7)	
H9	0.3492	0.0284	0.5689	0.079*	
C10	0.3458 (3)	−0.0965 (3)	0.4577 (2)	0.0798 (8)	
H10	0.3933	−0.1515	0.4927	0.096*	
C11	0.3032 (3)	−0.1225 (2)	0.3612 (2)	0.0713 (7)	
H11	0.3217	−0.1949	0.3306	0.086*	
C12	0.2334 (2)	−0.0415 (3)	0.3098 (2)	0.0632 (6)	
H12	0.2049	−0.0587	0.2438	0.076*	
C13	0.2049 (2)	0.0659 (2)	0.35541 (16)	0.0524 (5)	
H13	0.1574	0.1202	0.3195	0.063*	

### Atomic displacement parameters (Å^2^)

**Table:**

	*U*^11^	*U*^22^	*U*^33^	*U*^12^	*U*^13^	*U*^23^
Sn1	0.02733 (9)	0.03422 (10)	0.02977 (9)	−0.00209 (6)	0.00331 (6)	0.00222 (6)
Cl1	0.0462 (3)	0.0419 (2)	0.0420 (2)	−0.0050 (2)	0.00723 (19)	−0.00544 (19)
Cl2	0.0483 (3)	0.0508 (3)	0.0354 (2)	−0.0082 (2)	0.00231 (18)	0.00960 (19)
Cl3	0.0344 (2)	0.0550 (3)	0.0595 (3)	0.0041 (2)	0.0010 (2)	0.0053 (2)
N1	0.0344 (8)	0.0408 (8)	0.0418 (8)	−0.0066 (6)	0.0041 (6)	0.0081 (7)
C1	0.0606 (14)	0.0438 (11)	0.0561 (12)	−0.0041 (10)	0.0122 (10)	0.0042 (9)
C2	0.0721 (16)	0.0610 (15)	0.0650 (15)	0.0120 (13)	0.0182 (12)	0.0052 (12)
C3	0.0346 (10)	0.0675 (15)	0.0536 (12)	−0.0047 (9)	−0.0041 (9)	0.0039 (10)
C4	0.0640 (16)	0.101 (2)	0.0578 (14)	0.0305 (15)	−0.0091 (12)	0.0077 (14)
C5	0.0429 (11)	0.0497 (11)	0.0454 (10)	−0.0070 (9)	0.0037 (8)	0.0154 (9)
C6	0.0511 (13)	0.0902 (19)	0.0467 (12)	−0.0049 (12)	−0.0053 (10)	0.0124 (12)
C7	0.0375 (10)	0.0483 (11)	0.0453 (10)	−0.0073 (8)	0.0074 (8)	0.0075 (8)
C8	0.0348 (10)	0.0498 (11)	0.0443 (10)	−0.0025 (8)	0.0033 (8)	0.0071 (8)
C9	0.0628 (16)	0.0793 (19)	0.0554 (14)	0.0197 (13)	−0.0120 (12)	0.0016 (12)
C10	0.081 (2)	0.0746 (19)	0.084 (2)	0.0352 (16)	−0.0011 (15)	0.0087 (15)
C11	0.0777 (18)	0.0575 (15)	0.0793 (18)	0.0054 (13)	0.0182 (14)	−0.0086 (13)
C12	0.0640 (15)	0.0723 (16)	0.0535 (13)	−0.0050 (13)	0.0044 (11)	−0.0103 (12)
C13	0.0502 (12)	0.0615 (14)	0.0453 (11)	0.0037 (10)	−0.0008 (9)	0.0070 (10)

### Geometric parameters (Å, °)

**Table:**

Sn1—Cl3^i^	2.4207 (5)	C4—H4C	0.9600
Sn1—Cl3	2.4207 (5)	C5—C6	1.520 (3)
Sn1—Cl1	2.4237 (5)	C5—H5A	0.9700
Sn1—Cl1^i^	2.4237 (5)	C5—H5B	0.9700
Sn1—Cl2^i^	2.4579 (4)	C6—H6A	0.9600
Sn1—Cl2	2.4579 (4)	C6—H6B	0.9600
N1—C3	1.512 (2)	C6—H6C	0.9600
N1—C1	1.515 (3)	C7—C8	1.500 (3)
N1—C5	1.518 (2)	C7—H7A	0.9700
N1—C7	1.535 (2)	C7—H7B	0.9700
C1—C2	1.515 (3)	C8—C13	1.385 (3)
C1—H1A	0.9700	C8—C9	1.390 (3)
C1—H1B	0.9700	C9—C10	1.386 (4)
C2—H2A	0.9600	C9—H9	0.9300
C2—H2B	0.9600	C10—C11	1.364 (4)
C2—H2C	0.9600	C10—H10	0.9300
C3—C4	1.519 (3)	C11—C12	1.367 (4)
C3—H3A	0.9700	C11—H11	0.9300
C3—H3B	0.9700	C12—C13	1.384 (4)
C4—H4A	0.9600	C12—H12	0.9300
C4—H4B	0.9600	C13—H13	0.9300
			
Cl3^i^—Sn1—Cl3	180.0	H4A—C4—H4B	109.5
Cl3^i^—Sn1—Cl1	90.320 (18)	C3—C4—H4C	109.5
Cl3—Sn1—Cl1	89.680 (18)	H4A—C4—H4C	109.5
Cl3^i^—Sn1—Cl1^i^	89.680 (18)	H4B—C4—H4C	109.5
Cl3—Sn1—Cl1^i^	90.320 (18)	N1—C5—C6	115.36 (17)
Cl1—Sn1—Cl1^i^	180.0	N1—C5—H5A	108.4
Cl3^i^—Sn1—Cl2^i^	89.662 (17)	C6—C5—H5A	108.4
Cl3—Sn1—Cl2^i^	90.338 (17)	N1—C5—H5B	108.4
Cl1—Sn1—Cl2^i^	89.974 (17)	C6—C5—H5B	108.4
Cl1^i^—Sn1—Cl2^i^	90.026 (17)	H5A—C5—H5B	107.5
Cl3^i^—Sn1—Cl2	90.338 (17)	C5—C6—H6A	109.5
Cl3—Sn1—Cl2	89.662 (17)	C5—C6—H6B	109.5
Cl1—Sn1—Cl2	90.026 (17)	H6A—C6—H6B	109.5
Cl1^i^—Sn1—Cl2	89.974 (17)	C5—C6—H6C	109.5
Cl2^i^—Sn1—Cl2	180.00 (2)	H6A—C6—H6C	109.5
C3—N1—C1	111.75 (17)	H6B—C6—H6C	109.5
C3—N1—C5	106.60 (15)	C8—C7—N1	116.09 (15)
C1—N1—C5	110.92 (16)	C8—C7—H7A	108.3
C3—N1—C7	110.82 (15)	N1—C7—H7A	108.3
C1—N1—C7	106.10 (14)	C8—C7—H7B	108.3
C5—N1—C7	110.73 (15)	N1—C7—H7B	108.3
C2—C1—N1	115.49 (18)	H7A—C7—H7B	107.4
C2—C1—H1A	108.4	C13—C8—C9	117.5 (2)
N1—C1—H1A	108.4	C13—C8—C7	121.09 (19)
C2—C1—H1B	108.4	C9—C8—C7	121.3 (2)
N1—C1—H1B	108.4	C10—C9—C8	121.0 (2)
H1A—C1—H1B	107.5	C10—C9—H9	119.5
C1—C2—H2A	109.5	C8—C9—H9	119.5
C1—C2—H2B	109.5	C11—C10—C9	120.3 (3)
H2A—C2—H2B	109.5	C11—C10—H10	119.8
C1—C2—H2C	109.5	C9—C10—H10	119.8
H2A—C2—H2C	109.5	C10—C11—C12	119.7 (3)
H2B—C2—H2C	109.5	C10—C11—H11	120.1
N1—C3—C4	115.5 (2)	C12—C11—H11	120.1
N1—C3—H3A	108.4	C11—C12—C13	120.4 (2)
C4—C3—H3A	108.4	C11—C12—H12	119.8
N1—C3—H3B	108.4	C13—C12—H12	119.8
C4—C3—H3B	108.4	C8—C13—C12	121.1 (2)
H3A—C3—H3B	107.5	C8—C13—H13	119.4
C3—C4—H4A	109.5	C12—C13—H13	119.4
C3—C4—H4B	109.5		
			
C3—N1—C1—C2	58.9 (2)	C5—N1—C7—C8	59.4 (2)
C5—N1—C1—C2	−59.9 (2)	N1—C7—C8—C13	93.4 (2)
C7—N1—C1—C2	179.8 (2)	N1—C7—C8—C9	−91.3 (3)
C1—N1—C3—C4	52.4 (2)	C13—C8—C9—C10	−1.7 (4)
C5—N1—C3—C4	173.76 (19)	C7—C8—C9—C10	−177.2 (3)
C7—N1—C3—C4	−65.7 (2)	C8—C9—C10—C11	1.1 (5)
C3—N1—C5—C6	−174.68 (19)	C9—C10—C11—C12	0.0 (5)
C1—N1—C5—C6	−52.8 (2)	C10—C11—C12—C13	−0.5 (4)
C7—N1—C5—C6	64.7 (2)	C9—C8—C13—C12	1.2 (3)
C3—N1—C7—C8	−58.7 (2)	C7—C8—C13—C12	176.7 (2)
C1—N1—C7—C8	179.82 (17)	C11—C12—C13—C8	−0.1 (4)

Symmetry codes: (i) −*x*+1, −*y*+1, −*z*+1.

### Hydrogen-bond geometry (Å, °)

**Table:**

*D*—H···*A*	*D*—H	H···*A*	*D*···*A*	*D*—H···*A*
C2—H2B···Cl1^ii^	0.96	2.74	3.685 (3)	169

Symmetry codes: (ii) −*x*+1/2, *y*−1/2, −*z*+3/2.
